# The Influence of Heavy Metals and Trace Elements on Comatose Patients with Severe Traumatic Brain Injury in the First Week of Admission

**DOI:** 10.1155/2018/7252606

**Published:** 2018-09-17

**Authors:** Bahia Belatar, Abdallah Elabidi, Malika Barkiyou, Mamoun El Faroudi, Rachid Eljaoudi, Laila Lahlou, Saad Kabbaj, Wajdi Maazouzi

**Affiliations:** ^1^Research Unit of Cerebral Monitoring in Neuro-Reanimation, Faculty of Medicine and Pharmacy, University Mohammed V, Rabat, Morocco; ^2^Department of Toxicology, National Institute of Health, Rabat, Morocco; ^3^Laboratory of Histology, Embryology and Cytogenetic, Faculty of Medicine and Pharmacy, University Mohammed V and Emergency and Intensive Care Unit, Ibn Sina Hospital, Rabat, Morocco; ^4^Service of Anesthesiology and Reanimation, Ibn Sina University Hospital, Rabat, Morocco; ^5^Pharmacology and Toxicology Department, Faculty of Medicine and Pharmacy, University Mohammed V, Rabat, Morocco; ^6^Department of Social Medicine, Epidemiology & Public Health, Faculty of Medicine and Pharmacy, Rabat, Morocco; ^7^Service of Anesthesiology and Reanimation, Hospital of Specialties, Ibn Sina University Hospital, Rabat, Morocco

## Abstract

**Purpose:**

The aim of this study is to investigate the possible role of heavy metals (lead and cadmium) and imbalance of trace elements (chromium, iron, zinc, copper, and manganese) in death among patients with severe traumatic brain injury.

**Material and Methods:**

A case-control study was conducted with 64 comatose patients with severe TBI, in the Department of Anesthesiology and Reanimation, Ibn Sina University Hospital and Hospital of Specialties in Rabat, Morocco; 22 healthy volunteers were recruited in Blood Transfusion Center of Rabat. Blood samples were collected from TBI patients, in the first week (3h after admission and each 48h during one week) and from healthy volunteers one time. Concentration of heavy metals and trace elements in serum was determined by electrochemical atomic absorption spectrometry. Statistical analysis was performed using Statistical software (SPSS) and the cases and controls were compared using the Mann–Whitney U test and Student's* t*-test for cadmium according to gender and final evolution. A P-value <0.05 was considered to be statistically significant.

**Results:**

Our data showed that the difference of heavy metals concentration (lead and cadmium) between patients and healthy subjects was not statistically significant. However, the difference of some trace elements concentration (iron, copper, chromium, and selenium) between patients and healthy subjects was statistically significant. According to the final evolution, the concentration of manganese was higher in dead patients and statistically significant (p = 0.04) for heavy metals; the concentration of lead was not statistically significant while the concentration in cadmium was statistically significant (p = 0.004). By sex, lead and cadmium were statistically significant, respectively p = 0.02, p = 0.001, and cadmium was higher in women, while lead was higher in men.

**Conclusion:**

Among all studied heavy metals (lead and cadmium) and trace elements (iron, zinc, copper, selenium, chromium, and manganese), manganese and cadmium may play a role in the death of patients from severe traumatic brain injury.

## 1. Introduction

Heavy metals can be easily found in food and accumulated in organs and tissues after ingestion. Consequently, they create a significant threat to human health [1]. Exposure to some heavy metals such as lead and cadmium has particularly important effect on brain due to their long lasting [2]. Otherwise, chromium is used in many industries and it is widely distributed in the environment. Thus, the exposure to chromium dust has been reported among workers at these industries. In addition to its dangerous effects on organs, brain damage could be induced [3]. Trace elements, manganese, iron, copper, chromium, and zinc, are very important for cell functions at biological, chemical, and molecular levels. These elements mediate vital biochemical reactions by acting as cofactors for many enzymes, as well as acting as centers for stabilizing structures of enzymes and proteins [4]. Chromium is needed for biosynthesis of glucose tolerance factor. The deficiency causes impairment of glucose tolerance while toxicity results in renal failure, dermatitis, and pulmonary cancer [4]. Manganese is a vital nutrient in human body; it is an essential trace element maintained at an optimal level in human body for proper functioning of brain [5]; chronic exposure to manganese is associated with neurotoxicity and correlated with the development of various neurological disorders such as Parkinson's disease [5]. Iron is a microelement with the most completely studied biological functions; the biological role of iron is characterized by its indispensability in cell respiration and various biochemical processes providing normal functioning of cells and organs of the human body; iron also plays an important role in the generation of free radicals, which under different conditions can be useful or damaging to biomolecules and cells [6]; iron accumulation is a cardinal feature of degenerating regions in the Parkinson's disease brain [7], although an elevation in brain iron levels is a normal feature of ageing [7]. Copper is a biometal essential for normal brain development and function; however, copper deficiency or excess results in central nervous system diseases, such as disrupted Menkes disease and Wilson's disease, and neurodegenerative disorders, including Parkinson's disease, Alzheimer's disease, Amyotrophic lateral sclerosis, Huntington's disease, and prion diseases [8]. Zinc is abundant in the brain, where it plays an important role in synaptic plasticity and in learning, but excessive zinc is toxic to neuronal cells, which may lead to and Alzheimer's disease [9]. As regards traumatic brain injury (TBI), it is one of the leading causes of morbidity and mortality worldwide. In this context we aimed to investigate whether high level of heavy metals (lead and cadmium) and imbalance of trace elements (chromium, iron, zinc, copper, and manganese) could play a role in death among patients with severe traumatic brain injury.

## 2. Patients and Methods

### 2.1. Patients

#### 2.1.1. Study Design

This case-control study was conducted with 64 comatose patients with TBI identified as cases and 22 healthy volunteers identified as controls recruited in Blood Transfusion Center of Rabat.

#### 2.1.2. Selection Criteria of Cases and Controls

*Comatose Patients with Traumatic Brain Injury. *Patients included in this study were recruited during the period between December 2013 and January 2016 in the Department of Anesthesiology and Reanimation, Ibn Sina University Hospital and Hospital of specialties in Rabat, Morocco. We studied 64 comatose patients with severe traumatic brain injury (TBI): 57 men aged 42.7 ± 13.8 years and 7 women aged 39.00 ± 17.71 years.

*Inclusion Criteria. *Cases included in this study were comatose patients with severe TBI aged from 18 to 64 years. Neurological symptoms of severe TBI were confirmed by MRI/CT-scan.

*Exclusion Criteria. *Patients excluded from the study were smoker, alcoholic, and receiving barbiturates medication, corticoids, or any treatment that could have an antioxidant effects and patients who underwent a craniotomy, with febrile state and/or septic shock state and psychological problems.

#### 2.1.3. Healthy Volunteers

22 Healthy volunteers did not show clinical symptoms or signs of any diseases. All participants were mentally and physically able to participate in the study. The age of volunteers was matched with the age of patients. The study protocol was approved by a local ethics committee for biomedical research in Rabat, Morocco. Healthy volunteers have read and signed a consent form before being included in the study.

### 2.2. Methods

4ml of peripheral venous blood was collected in plasma tube. Blood sampling in TBI patients was performed within the first week (3h after admission during one week). Blood samples were centrifuged at 4.000rpm/min for 15 min at 4°C. Then serum samples were aliquoted and stored at -80°C for later determination heavy metals (lead and cadmium) and trace elements (iron, copper, zinc, chromium, and manganese). 1ml of plasma was taken from each sample and digested by 0.5ml of nitric acid 65% (Merck) in a polyethylene tube, which was very well closed and put in the Bath of Marie at 120° for 4 hours, until the solution became clear and no fumes were observed; each sample was then diluted to 10 ml with doubly distilled water [10]. The dosage of Pb, Cu, Mn, Cr, and Cd in blood samples was accomplished by (VARIAN GTA 120 AA240Z) atomic absorption spectroscopy with graphite furnace; the background correction was made by a Zeeman effect, while the dosage of Fe and Zn was performed by atomic absorption spectroscopy (VARIAN AA40 FS) with flame, in the accredited laboratory of toxicology in the National Health Institute (Rabat, Morocco). To reduce chemical interferences and volatility of Cd and Pb in a furnace, a matrix modifier was used (mixture of PdCl2 and MgNO3). The calibration curve was made by the “MSA (Method of Standard Addition)” [11].

### 2.3. Results

Clinical data collected from medical records (Table 1) showed that most of patients with severe traumatic brain injury are men while women proportion represents 10.9 %, and the death rate among patients in the first week of hospitalization was nearly 60%. Additionally, the death rate in men was significantly higher (63.2%) while in women the death rate was 28.6%.

We compared the concentration of prognostic factors collected from medical record in dead patients and surviving patients. Data are summarized in Table 2. All clinical parameters including arterial pressure of CO2 and O2, cardiac frequency, activated partial thromboplastin time, fraction of inspired oxygen, Red Blood Cells in first, second, and third day of hospitalization, leukocytes in first and second day, Hematocrit, and Thrombocytes were not statistically significant in patients except the volume of Hematocrit of the first day of hospitalization.

We studied the concentration of heavy metals and oligo-elements between patients and healthy volunteers, and our data showed that the difference of heavy metals concentration (lead and cadmium) between patients and healthy subjects was not statistically significant. However, the difference of some trace elements concentration (iron, copper, chromium, and selenium) between patients and healthy subjects was statistically significant, while manganese and zinc concentrations were not significantly higher (Table 3).

Data of comparison of heavy metals and trace elements according to the survival and nonsurvival status are summarized in Table 4(b). The concentration of iron and zinc was not statistically significant among the two groups of patients in the first, the second, and the third day. The same data was remarked in copper and chromium concentration (p>0.05). Manganese concentration was, however, higher in dead patients and statistically significant (p=0.04); As regards heavy metals, lead concentration was not statistically significant while the concentration of cadmium was higher in surviving patients with p=0.004 (Student's* t*-test, Table 4(a)).

According to Table 5. All studied trace elements were not statistically significant according to gender. Besides, concentration of heavy metals, lead and cadmium, was statistically significant and cadmium was higher in women while lead was higher in men.

## 3. Discussion

According to our clinical data, most of patients with severe traumatic brain injury are men and the death rate among patients in the first week of hospitalization was nearly 60%. Additionally, the death rate in men was significantly higher compared to women. Many findings support our data; some authors reported an increased level of mortality with age and gender; higher level in men with TBI was found [12–14].

The Glasgow Coma Scale scores were collected in three days within admission. In fact, all patients in the first day showed severe score of GCS. In the third day after admission, 90.9% had severe GCS score compared to 9.1% of moderate scores. In the fifth day GCS score was also 81.81% severe and 18.19% moderate [15].

As regards prognostic factors including arterial pressure of CO2 and O2, cardiac frequency, activated partial thromboplastin time, fraction of inspired oxygen, Red Blood Cells in first, second, and third day of hospitalization, leukocytes in the first and the second day, Hematocrit, and Thrombocytes were not different in patients except the volume of Hematocrit of the first day of hospitalization. Similarly, the major determinants of disability and death in patients with traumatic brain injury according to many authors, intracranial haemorrhage, prothrombin time, anaemia and platelets, and haemoglobin, are all associated with the poorer outcome in patients after TBI [16–18].

Heavy metals concentration (cadmium and lead) in our study were not elevated in patients compared to healthy subjects. Additionally, comparison of lead and cadmium concentration in dead and surviving patients showed no difference between groups of patients in lead concentration while the concentration of cadmium was statistically significant (p=**0.004**). Regarding gender, our data showed that cadmium was higher in women while lead was higher in men. It has been shown that chronic exposure of mice to cadmium induced brain damage or neuronal cell death [19]. Also, lead is one of the most common environmental toxicants associated with decline in brain function [4]. Cd and Pb are among the heavy metals of greatest concern for health; for comparing the effect of these metals, two different sites at the level of the respiratory system, H441 and alveolar bronchiolar cells A549, were used; these lines are used enough to study the transport and toxicity of several metals; other studies find that the effect of ligands involved in cytoprotection against Cd toxicity indicates that Cd-resistant cells have a GSH level twice as high as sensitive cells. For the cytotoxicity of Cd and Pb, the exposure of A549 and H441 cells in Pb in serum-free culture medium practically did not induce cell death; Pb had no effect on cell viability in A549 and H441 cells; in both cell lines (A549 and H441), Cd is cytotoxic, whereas Pb does not affect cell viability. However, the A549 line appears to be twice as sensitive to Cd as H441 cells, and Cd cell accumulation studies indicate that the difference of this sensitivity between the lines is not related to a greater accumulation of Cd but rather attributable to other cellular protection mechanisms such as the presence and/or induction of MTs, GSH, or HSP70. It would therefore be relevant to compare the antioxidant capacity as well as the induction levels of these stress proteins in cells A549 and H441 [20].

Otherwise, in the studied trace elements, iron, zinc, copper, selenium, chromium, and manganese, some elements showed a difference between patients and healthy subjects (iron, copper, and chromium) while manganese and zinc concentration were not different between them; in dead and surviving patient, there were no differences of trace elements concentration except manganese concentration (p=**0.04**) that was, however, higher in dead patients. While comparing concentration of trace elements by gender, the data did not reveal any difference between men and women; it is known that elevated manganese levels can cause human neurotoxicity [21]. Traumatic patients develop an acute phase response characterized by decreased levels of some elements such as iron and zinc and increased levels of others like copper [22–25]. Some authors reported an increased serum concentration of chromium in patients with head injury; however, the concentration of chromium in serum had no significant relation to the degree of head injury during the first 4 hours [22, 25]. Some studies have identified increased toxic levels of zinc after experimental injury; however, others have reported zinc deficiency as a major problem after TBI and demonstrated zinc supplementation to be an effective therapy [26, 27]. Moreover, TBI studies have linked zinc accumulation with deterioration of nerve cell [28, 29]. Copper levels in a study conducted among patients with traumatic brain injury showed higher levels in patients compared to controls. Moreover, the difference between the moderate and severe traumatic brain injury patients was insignificant [30].

In conclusion, among all studied heavy metals (lead and cadmium) and trace elements (iron, zinc, copper, chromium, and manganese), manganese and cadmium may play a role in the death of patients from severe traumatic brain injury.

## Figures and Tables

**Table 1:** Clinical data collected from medical records of patients with Severe Traumatic Brain Injury within one week in admission.

**Clinical data of patients**	
**Sex percentage (%) **	
Women (n=7)	10.9
Men (n=57)	89.1
**Death rate within one week (%) (n=38)**	59.4
**Survival rate within one week (%) (n=26)**	40.6
**Survival rate in men (%)**	
Dead patients (n=36)	63.2
Survived patients (n=21)	36.8
**Survival rate in women (%)**	
Dead patients (n=2)	28.6
Survived patients (n=5)	71.4

**Table 2:** Prognostic factors collected from medical record according to death and survival status.

**Clinical parameters**	**Dead patients**	**Survived patients**	**P value**
**PaCO2 (mmHg)**	35.5 ± 4.76 (n=14)	34.8 ± 3.42 (n=5)	0.44
**PaO2 (mmHg)**	191.58 ± 97.04 (n=12)	156 ± 68.07 (n=5)	0.13
**CF (bpm)**	87.89 ± 20,03 (n=28)	80.11 ± 32.52 (n=18)	0.09
**APTT (s)**	28.28 ± 7.34 (n=19)	29.68 ± 5.53 (n=8)	0.93
**FiO2 (L/min)**	55.95 ± 13.19 (n=21)	52.81 ± 14.13 (n=16)	0.79
**RBC (×10** ^**6**^/**µl) day 1**	3.78 ± 0.71 (n=23)	4.12 ± 0.54 (n=14)	0.27
**RBC (×10** ^**6**^/**µl) day 2**	3.79 ± 0.67 (n=23)	3.92 ± 0.28 (n=11)	0.54
**RBC (×10** ^**6 **^/**µl) day3**	3.83 ± 0.72 (n=4)	3.93 ± 0.3 (n=2)	0.33
**Leukocytes (×10** ^**3**^/**µl) day 1**	11.35 ± 2.24 (n=23)	12.37 ± 1.8 (n=14)	0.32
**Leukocytes (×10** ^**3**^/**µl) day 2**	10.9 ± 2.26 (n=12)	11.5 ± 1.7 (n=5)	0.18
**HCT (vol**%**) day 1**	31.63 ± 9.1 (n=24)	36.42 ± 4.69 (n=14)	**0.04**
**Thr (10** ^**3**^ **/mm** ^**3**^ **) day1**	173.69 ± 87.4 (n=23)	195.28 ± 64.06 (n=14)	0.23
**Thr (10** ^**3**^ **/mm** ^**3**^ **) day2**	139.58 ± 84.71 (n=12)	229.8 ± 95.72 (n=5)	0.47

**CF**: cardiac frequency; **PaO2:** Arterial oxygen partial pressure; **PaCO2**: Arterial carbon dioxide partial pressure; **APTT:** activated partial thromboplastin time; **FiO2:** Fraction of inspired oxygen; **RBC**: Red Blood Cells; **HCT**: Hematocrit; **Thr: **Thrombocytes.

**Table 3:** Heavy metals and trace elements concentration in patients and healthy volunteers (Cr, Cd, Pb, Mn, Cu,, the data show an average over the period of 48H).

**Heavy metals and trace elements concentration **	**Patients**	**Healthy volunteers**	**P value**
**Chromium (** **µ** **g/L)**	240.07 (162.30; 616.52)	4835.25 (240.93; 53385.49)	**0.008**
**Cadmium (** **µ** **g/L)**	9.94 (9.43; 10.14)	9.46 (0.95; 10.02)	0.33
**Lead (** **µ** **g/L)**	46.28 (24.25; 107.67)	48.93 (11.52; 127.37)	0.54
**Manganèse (** **µ** **g/L)**	47.77 (14.15; 75.99)	74.10 (22.23; 955.25)	0.10
**Copper (** **µ** **g/L)**	1065.58 (655.63; 1627.95)	2431.32 (1484.82; 3352.30)	**0.01**
**Zinc (mg/L)**	0.0 (0.00; 0.00)	0.00 (0.00; 0.38)	0.12
**Iron (mg/L)**	0.0 (0.00; 0.00)	6.52 (2.59; 56.72)	**0.00**
**Sélénium (** **µ** **g/L)**	107.08 (15.22; 350.50)	34.00 (28.00; 46.00)	**0.03**

**Table:** (a) Cadmium concentration in dead and survived patients.

**Heavy metals concentration**	**Dead patients **	**Survived patients **	**P value**
**Cadmium (** **µ** **g/L)**	6.78 ± 4.85 (n=23)	10.08 ± 0.47 (n=14)	**0.004**

**Table:** (b) Heavy metals and trace elements concentration in dead and survived patients.

**Heavy metals concentration**	**Dead Patients**	**Survived patients**	**P value**
**Se d0 (** **µ** **g/L)**	141.47 (33.43; 400.35)	75.00 (3.77; 3 17.90)	0.58
**Se d2 (** **µ** **g/L)**	308.60 (8.95; 729.40)	329.20 (82.57; 557.85)	0.96
**Se d4 (** **µ** **g/L)**	274.30 (85.00; 494.50)	104.80 (0.00; 173.68)	0.10
**Iron d0 (mg/L)**	0.00 (0.00; 3.17)	0.00 (0.00; 3.72)	0.75
**Iron d2 (mg/L)**	0.00 (0.00; 2.18)	3.90 (0.00; 7.56)	0.63
**Iron d4 (mg/L)**	0.43 (5.73; 0.00)	0.83 (0.00; 0.86)	0.62
**Zinc d0 (mg/L)**	0.00 (0.00; 0.00)	0.00 (0.00; 0.00)	0.75
**Zinc d2 (mg/L)**	0.00 (0.00; 0.00)	0.00 (0.00; 0.00)	0.97
**Zinc d4 (mg/L)**	0.00 (0.00; 0.00)	0.00 (0.00; 0.00)	0.23
**Cu (** **µ** **g/L)**	949.88 (716.09; 1422. 90)	1323.51 (627.82; 1943.76)	0.42
**Cr (** **µ** **g/L)**	219.97 (147.57; 572.37)	260.10 (171.14; 620.64)	0.45
**Pb (** **µ** **g/L)**	42.52 (15.38; 130.46)	51.75 (28.31; 107.44)	0.56
**Mn (** **µ** **g/L)**	21.15 (4.87; 58.77)	58.60 (35.82; 78.44)	**0.04**

**Table 5:** Heavy metals concentration according to the gender.

**Heavy metals concentration**	**Men**	**Women**	**P value**
Cadmium (*µ*g/L)	7.61 ± 4.33 (n=43)	9.95 ± 0.34 (n=5)	**0.001**
Lead (*µ*g/L)	51.75 (23.94; 117.6) (n=53)	11.52 (0; 42.98) (n=5)	**0.02**

## Data Availability

The data used to support the findings of this study are available from the corresponding author upon request.

## References

[1] Xu M., Wang P., Sun Y., Yang L., Wu Y. (2017). Joint toxicity of chlorpyrifos and cadmium on the oxidative stress and mitochondrial damage in neuronal cells. *Food and Chemical Toxicology*.

[2] Karri V., Schuhmacher M., Kumar V. (2016). Heavy metals (Pb, Cd, As and MeHg) as risk factors for cognitive dysfunction: A general review of metal mixture mechanism in brain. *Environmental Toxicology and Pharmacology*.

[3] Salama A., Hegazy R., Hassan A. (2016). Intranasal chromium induces acute brain and lung injuries in rats: Assessment of different potential hazardous effects of environmental and occupational exposure to chromium and introduction of a novel pharmacological and toxicological animal model. *PLoS ONE*.

[4] Chitturi R., Baddam V. R., Prasad L., Prashanth L., Kattapagari K. (2015). A review on role of essential trace elements in health and disease. *Journal of Dr. NTR University of Health Sciences*.

[5] Tarale P., Chakrabarti T., Sivanesan S., Naoghare P., Bafana A., Krishnamurthi K. (2016). Potential Role of Epigenetic Mechanism in Manganese Induced Neurotoxicity. *BioMed Research International*.

[6] Milto I. V., Suhodolo I. V., Prokopieva V. D., Klimenteva T. K. (2016). Molecular and cellular bases of iron metabolism in humans. *Biochemistry (Moscow)*.

[7] Hare D. J., Double K. L. (2016). Iron and dopamine: A toxic couple. *Brain*.

[8] Davies K. M., Mercer J. F., Chen N., Double K. L. (2016). Copper dyshomoeostasis in Parkinson's disease: implications for pathogenesis and indications for novel therapeutics. *Clinical Science*.

[9] Li L. B., Wang Z. Y. (2016). Disruption of brain zinc homeostasis promotes the pathophysiological progress of Alzheimer's disease. *Histol Histopathol*.

[10] Auger D. Méthode de dosage des métaux traces dans les milieux biologiques. *Direction de l’environnement et de recherches océaniques*.

[11] Bounagua M., Bellaouchou A., Benabbou A., El Abidi A., Ben-aakam R., Fekhaoui M. (2014). Using bloods Passer domesticus as a possible bio-indicator of urban heavy metals pollution in Rabat-Salé (Morocco). *Journal of Materials and Environmental Science*.

[12] Taylor C. A., Bell J. M., Breiding M. J., Xu L. (2017). Traumatic brain injury-related emergency department visits, hospitalizations, and deaths - United States, 2007 and 2013. *MMWR Surveillance Summaries*.

[13] Nyam T. E., Ao K., Hung S., Shen M., Yu T., Kuo J. (2017). FOUR Score Predicts Early Outcome in Patients After Traumatic Brain Injury. *Neurocritical Care*.

[14] Ha M., Kim B. C., Choi S., Cho W. H., Choi H. J. (2016). Preventable and Potentially Preventable Traumatic Death Rates in Neurosurgery Department: A Single Center Experience. *Korean Journal of Neurotrauma*.

[15] Belatar B., Laidi F., El Abidi A. (2018). Serum levels of selenium and C-reactive protein in comatose patients with severe traumatic brain injury during the first week of hospitalization: Case-control study. *Pan African Medical Journal*.

[16] Kulesza B., Nogalski A., Kulesza T., Prystupa A. (2015). Prognostic factors in traumatic brain injury and their association with outcome. *Journal of Pre-Clinical and Clinical Research*.

[17] Van Beek J. G. M., Mushkudiani N. A., Steyerberg E. W. (2007). Prognostic value of admission laboratory parameters in traumatic brain injury: results from the IMPACT study. *Journal of Neurotrauma*.

[18] Joseph B., Aziz H., Zangbar B. (2014). Acquired coagulopathy of traumatic brain injury defined by routine laboratory tests: which laboratory values matter?. *Journal of Trauma and Acute Care Surgery*.

[19] Chen S., Ren Q., Zhang J. (2014). N-acetyl-L-cysteine protects against cadmium-induced neuronal apoptosis by inhibiting ROS-dependent activation of Akt/mTOR pathway in mouse brain. *Neuropathology and Applied Neurobiology*.

[20] Mantha M., El Idrissi L., Leclerc-Beaulieu T., Jumarie C. (2011). Fe- and Zn-induced inhibition of Cd uptake in human lung cell lines: Speciation studies with H441 and A549 cells. *Toxicology in Vitro*.

[21] Neal A. P., Guilarte T. R. (2015). Mechanisms of Heavy Metal Neurotoxicity: Lead and Manganese. *Journal of Drug Metabolism & Toxicology*.

[22] Eskandary H., Nowbari N. (2002). Serum concentration of chromium in head injury patients. *Archives of Iranian Medicine*.

[23] Young A. B., Ott L. G., Beard D., Dempsey R. J., Tibbs P. A., McClain C. J. (1988). The acute-phase response of the brain-injured patient. *Journal of Neurosurgery*.

[24] McClain C. J., Twyman D. L., Ott L. G. (1986). Serum and urine zinc response in head-injured patients. *Journal of Neurosurgery*.

[25] Berger M. M., Shenkin A. (1998). Trace elements in trauma and burns. *Current Opinion in Clinical Nutrition & Metabolic Care*.

[26] Frederickson C. J., Cuajungco M. P., Frederickson C. J. (2005). Is zinc the link between compromises of brain perfusion (excitotoxicity) and Alzheimer's disease?. *Journal of Alzheimer's Disease*.

[27] Frederickson C. J., Maret W., Cuajungco M. P. (2004). Zinc and Excitotoxic Brain Injury: A New Model. *The Neuroscientist*.

[28] Hellmich H. L., Eidson K. A., Capra B. A. (2007). Injured Fluoro-Jade-positive hippocampal neurons contain high levels of zinc after traumatic brain injury. *Brain Research*.

[29] Suh S. W., Chen J. W., Motamedi M. (2000). Evidence that synaptically-released zinc contributes to neuronal injury after traumatic brain injury. *Brain Research*.

[30] Johary A., Vinod J., Misra S., Vibhuti R., Singh J. V. (2014). Impact of Serum Copper Level in Patients of Traumatic Brain Injury and its Correlation with Glasgow Coma Scale. *International Journal of Science and Research (IJSR)*.

